# Development and *in vitro* Evaluation of Enteric Coated Multiparticulate System for Resistant Tuberculosis

**DOI:** 10.4103/0250-474X.44597

**Published:** 2008

**Authors:** Md. A. Rahman, J. Ali

**Affiliations:** Department of Pharmaceutics, Faculty of Pharmacy, Jamia Hamdard, (Hamdard University), New Delhi-110 062, India

**Keywords:** Pellets, extrusion/spheronization, Eudragit L 30 D55, fluidized bed coater, enteric-coated

## Abstract

The multiparticulate formulation of sodium para aminosalicylate for oral administration was developed by extrusion spheronization technique. Microcrystalline cellulose was used as filler in concentration of 14.4% w/w. Pellets were coated with Eudragit L 30 D-55 using fluidized bed processor. Different weight gains of acrylic polymer were applied onto the pellets and evaluated for *in vitro* dissolution behavior in 0.1 N HCl for two hours and then media was changed to phosphate buffer pH 6.8. A 60% w/w coating level of Eudragit L30 D 55 has produced the most acceptable results against the gastric attack. 3% Seal coat of HPMC E5 was also applied in order to protect the drug from migration into the Eudragit coat and film coat was applied in order to prevent aggregation of pellets in the dissolution media. Morphological characteristics of developed pellets were also investigated by scanning electron microscopy and found to be smooth and spherical. Developed system was found to be suitable for the delivery of Sod PAS in to intestinal region.

Sodium para aminosalicylate (sodium-4-amino-hydroxy benzoate dehydrate, Sod PAS) is a bacteriostatic agent against the mycobacterium tuberculosis and frequently prescribed for treatment of resistant tuberculosis at a very high dose (12-15 g). Sod PAS is highly gastric irritant. The gastrointestinal side effects are commonly seen when drug is administered orally and 10% of the drug is degraded in gastric acidic environment to form 3-aminophenol, which is hepatotoxic in nature. In order to eliminate these adverse effects and to prevent degradation of drug at acidic pH, enteric coated product has been aimed.

The focus of the present study was to produce pellets as multi-particulate delivery system, because of its advantages over monolithic dosage forms. Particle less than 2-3 mm can rapidly pass the pylorus regardless of the filling level of the stomach as well as on size and density of chyme^1^ also gastrointestinal irritations are reduced by spreading in the intestine. A burst effect has so far not been reported with multiparticulate systems. Microcrystalline cellulose is used as universal filler and binder for the pellets formation by extrusion and spheronization.

The enteric polymer, Eudragit L30 D 55 is anionic copolymer contained free carboxylic groups in ratio of 1:1 with the ester groups. The carboxylic groups ionize in aqueous media at pH 5.5 and above, rendering the polymer resistant to the acidic environment of the stomach, but soluble at intestinal pH^2^–^3^. Fluidized bed coater performed enteric coating. The fluid bed coating is currently a widely used technique because it allows, among the other applications, crystals or granules to be coated with a variety of available polymers to provide gastro-resistant or controlled release system.

Highly water-soluble drugs require higher polymer coating levels than poorly soluble compounds for sustained or delayed drug release. Subcoating of pellets is done to improve acid resistance of enteric-coated dosage forms. Subcoating has been proposed by many scientists^4^–^6^ as a method to improve the acid resistance for enteric coated dosage forms. Polymeric subcoats functions by sealing the substrate from the aqueous enteric film coating, thus preventing the migration of water soluble drugs in to polymeric film, as well as preventing drug polymer interaction. The aim of this study was to develop an enteric coated multiparticulate formulation, which ensures the protection of sod PAS in acidic environment and delivers the drug in intestinal region.

## MATERIALS AND METHODS

Sod PAS was a gift sample by Lupin Ltd., Pune, India. Following materials were used for the development of multiparticulate system: microcrystalline cellulose PH 101 (Ran Q Remedies Ltd., India), cross povidone NF (ISP Chemicals, India), HPMC E5 (Dow Chemicals, India), sodium metabisulphite (Milan Chemical Ltd., India), corn starch (Neshiel Chemical Ltd., India), Eudragit L 30D-55 (Rohm Pharma, India), simethicone emulsion USP (Dow Cornin., India). All the reagents and solvents used were of analytical grades.

### Preparation of pellets:

 The core pellets contained 80% w/w Sodium para aminosalicylate, 14.4% w/w MCC RANQ PH101, 2.5% w/w cross povidone, 2% w/w corn starch, 1% w/w HPMC E5 and 0.1% w/w sodium metabisulphite. The dry ingredients were combined and mixed in twin-shell blender for 20 min and then transferred to planetary mixture where the aqueous binder solution prepared with HPMC E5, was slowly added. The wet mass was extruded with a Fauji Paudal granulator through a 1.2 mm screen. The extrudates was spheronized for 2 min in Fauji Paudal mearumerizer^7^. The pellets were dried at 40° for half an hour then drying was continued at 60° and then sized through 16-25 mesh screen. Processing parameters are given in Table 1.

**TABLE 1:** PROCESS CONDITIONS AND PARAMETERS USED FOR OPTIMIZED PELLETS

Parameters	Value
Spheronization speed	1100 rpm
Spheronization load	200 g
Spheronization time	2 minute
Amount of granulation liquid	65 ml
Screen used	1.2 mm
Drying temperature	40-60 °

Process conditions and parameters used for preparation of 250 g batch of optimized pellets

### Size distribution of uncoated pellets:

Size of the pellets was determined by sieve shaker analysis. Sieves of different sizes including, # 30 (590), # 25 (710 μm), # 20 (840 μm), # 18 (1000 μm), # 16 (1190 μm) and # 14 (1410 μm) were placed on mechanical shaker. Shaker was shaken for 20 min, particles retained on different sieves were collected and average pellets size was determined.

### Pellet friability:

The friability of the uncoated pellets was determined by inserting 20 g sample of pellets inside a fluidized bed unit fitted with a Wurster insert. The pellets were fluidized for 15 min. at 30°. Pellets were then transferred from the fluidized bed and the residual powder was removed prior to recording the final weight. The percentage friability was calculated from the pellet weight before and after fluidization^8^.

### Angle of repose:

 The angle of repose was measured using funnel with 6 mm diameter orifice 20 g of pellets were placed in funnel and allowed to fall 4 cm onto a hard level surface. The repose angle was determined by height and radius of the resulting pellets.

### Aerated bulk density of uncoated pellets:

 Twenty grams of pellets were poured gradually through a funnel in to 50 ml graduated cylinder, tapped lightly on hard surface and the volume measured. Bulk density was calculated as the quotient of the weight and volume of pellets.

### Packed bulk density of uncoated pellets:

 Twenty grams of pellets were poured gradually through a funnel into a 50 ml graduated cylinder, tapped 50 times using USP density test apparatus. The packed bulk density was calculated as the quotient of the weight and volume sedimented.

### Carr’s index and Hausner’s ratio:

The flowability of developed pellets was determined by the Carr’s index and Hausner’s ratio calculated using the following formulae,

$$\begin{array}{l} \text{Carr’s index}=\frac{\text{Tapped density}-\text{Bulk density}}{\text{Tapped density}}\times 100\\ \text{and}\\ \text{Hausner’s ratio}=\frac{\text{Tapped density}}{\text{Tapped density.}}\times 100 \end{array}$$

### Enteric coating of drug loaded pellets, seal coat:

 Talc (7.75% w/w) and titanium oxide (2.58% w/w) were dispersed in a mixture of solvents (isopropyl alcohol and methylene chloride, 1:1.5) and then passed through colloid mill for 5 minute. HPMC E5 (68.21% w/w) and ethyl cellulose (7.23% w/w) were dissolved in it with stirring, PEG 6000 (9.04% w/w) and propylene glycol (5.16% w/w) were added as plasticizer.

### Enteric coating of drug loaded pellets:

 The acrylic polymer coating suspensions were prepared by adding water to the commercially available Eudragit L30 D 55. Sod PAS containing pellets 16-25 mesh were coated with the aqueous coating dispersion in a fluid-bed coater. A 200 g of batch of pellets was placed in the fluid bed coater and pre warmed for 10 min prior to the spraying. Ten percent aqueous solution of PEG 6000 (8.29% w/w) containing 8.29% w/w of talc and 0.51% of simethicone was mixed with 82.89% w/w dispersion of Eudragit L 30 D. A peristalsis pump was used to deliver the coating dispersion to the spray nozzle. A magnetic stirrer was used to continually mix the coating dispersion during the coating process to prevent sedimentation. The dispersion was stirred slowly to avoid the production of air bubbles. Typical processing parameters for the film coating are listed in Table 2. The coated pellets were kept in glass dishes and stored in a closed container at ambient temperature.

**TABLE 2:** PROCESS CONDITIONS FOR COATING OF PELLETS

Parameters used	Condition		
	
	Seal coat	Eudragit coat	Film coat
Heater temperature (°)	65	65	60
Inlet temperature (°)	55	50	50
Outlet temperature (°)	45	40	40
Product bed temperature (°)	37	30	30
Atomization pressure (kg/cm^2^)	1.2-1.5	1.2-1.5	1.2
Needle (kg/cm^2^)	2	2	2
Exhaust air velocity Ft3/min	42	42	42
Peristaltic pump (rpm)	6-10	4-8	6-12
Blower (HZ)	17-23	17-25	17-25

An over coat was added to Sod PAS pellets that were coated with Eudragit L 30 D to prevent the sticking of the pellets during dissolution studies and storage. Glcerylmonostearate dispersion in methylene chloride was used for this purpose and was prepared by adding the powder to the solvent. This dispersion were then applied to the pellets resulting in 3% weight gain after the Eudragit coat sprayed up to 36%, 46% and 56% for 40%, 50% and 60% w/w level.

### *In vitro* release behavior:

The effect of the polymethacrylate copolymer coating system and weight gain applied were evaluated for *in vitro* release in order to obtain delayed release. The United States Pharmacopoeia USP 24 I (basket) method was used to investigate the dissolution properties of Sod PAS from coated pellets in 900 ml of medium, (0.1 N HCl for 2h then media was changed to phosphate buffer pH 6.8) maintained at 37° over a 12 h period. Media were agitated at 100 rpm and samples were taken at regular intervals, filtered through 0.45 μm cellulose fibers, analyzed spectrophotometrically at 300 nm for 0.1 N HCl and 265 nm for phosphate buffer for sod PAS content. The cumulative percent drug release-time profiles were determined.

### Microscopic studies:

 Shape and surface characteristics were determined by scanning electron microscope using gold sputter technique. The particles were vacuumed, dried and coated with gold palladium and observed microscopically.

## RESULTS AND DISCUSSION

As the drug is highly gastric irritant and about 10% drug gets degraded in acidic environment, the present study was focused to prevent the acidic degradation of Sod PAS in upper gastrointestinal tract. System was based on enteric coated multiparticulate units filled in hard gelatin capsules.

In preliminary trials, various formulation combinations (Table 3) and conditions including spheronization speed, amount of granulating liquid, and spheronization time were investigated to produce spherical and smooth surface pellets (Table 2). Optimized process conditions and parameters are listed in Table 1. Microcrystalline cellulose PH 101 has been found to be an effective diluent in extrusion spheronization. With increase in MCC content, surface of pellets had more plastic nature due to accommodate an increased granulating liquid thus they could be easily deformed during spheronization. On the basis of physical properties and dissolution behavior formulation F4 was taken as optimized formulation. A 14.4% w/w content of MCC was found to be optimum to produce the pellets of required properties. Similarly pellets were also prepared with varying amount of granulating liquid, higher granulating liquid content resulted in harder pellets that could be a factor in delaying dissolution behavior. Sixty five milliliters of granulating liquid (water) was found to produce pellets with optimum hardness. The total out put of extrudate is mainly governed by the extrusion speed. At lower speed (830 rpm) dumbbell shaped and irregular pellets were observed. A speed of 1100 rpm was selected as optimized speed for the production of spherical and smooth surface pellets. Addition of 2% w/w corn starch further improved the smoothness of surface. Drug turns brown when comes in contact with air due to oxidation of phenolic group in benzene moiety, therefore 0.1% sodium metabisulphite were added as antioxidant. Average pellet size was determined by sieve analysis and found to be 860.66 μm.

**TABLE 3:** FORMULAE FOR DIFFERENT PELLETS

Ingredients	Formulations			
	
	F1 %	F2 %	F3 %	F4 %
Sod PAS	80	80	80	60
MCC RAN Q PH101	14.4	15.4	16.4	14.4
Cross povidone	2.5	2.5	2.5	2.5
Corn starch	-	-	-	2.0
HPMC E5	3.0	2.0	1.0	1
Sodium metabisulphite	0.1	0.1	0.1	0.1

**Fig. 1:**
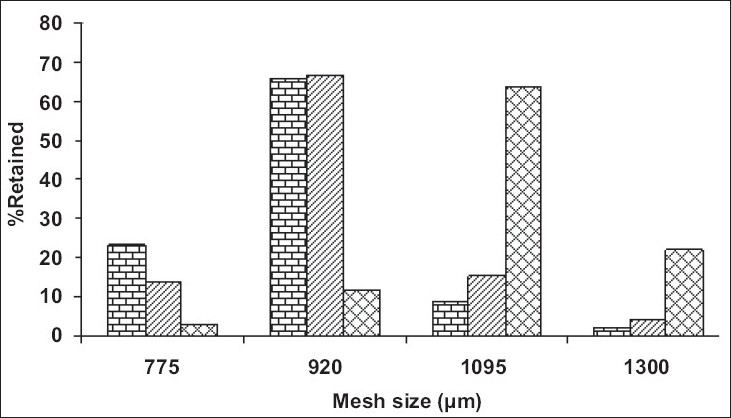
Size distribution of coated pellets Size distribution of coated pellets with 40% (
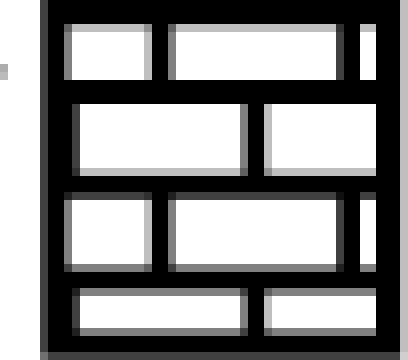
), 50% (
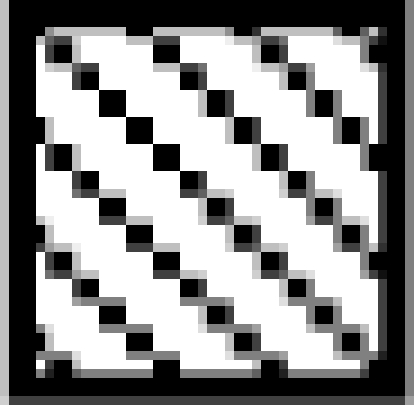
) and 60% (
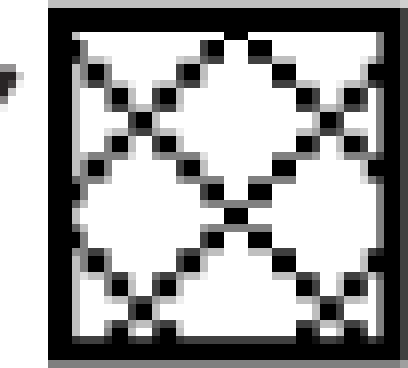
) coating

**Fig. 2:**
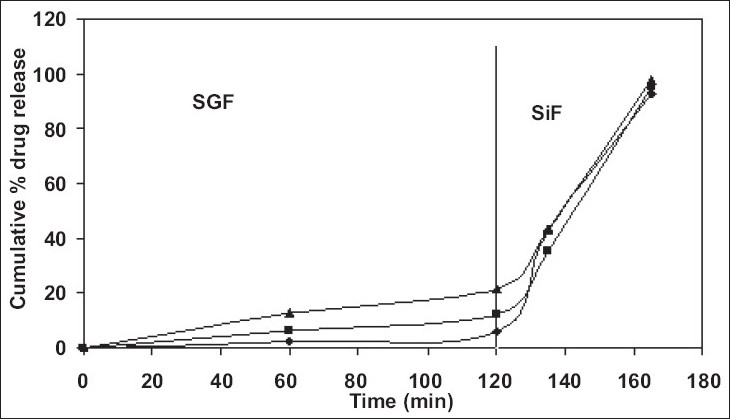
*In vitro* release of sod PAS from coated pellets with different weight gain. Release of sodium para aminosalicylate (sod PAS) *in vitro* in simulated gastric (SGF) and intestinal fluids (SIF) from coated pellets containing 40% (–♦–), 50% (–■–) and 60% (–▲–) coats

**Fig. 3:**
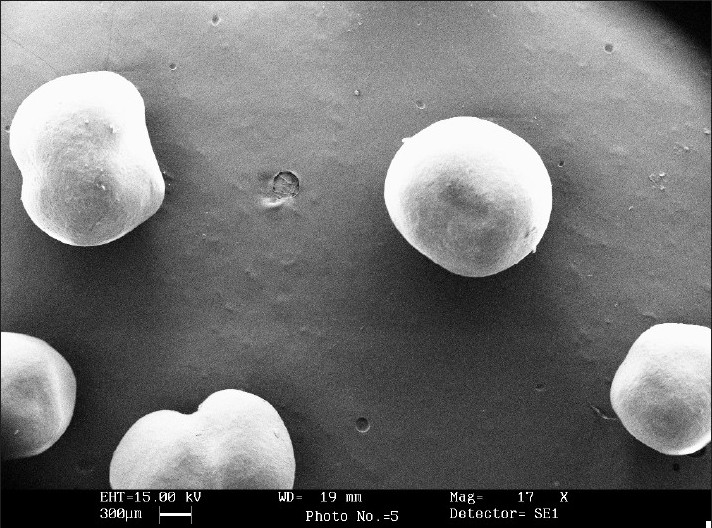
Scanning electron micrograph of developed pellets

In order to avoid the gastric irritation and degradation of drug in upper gastro intestinal tract, above developed pellets were further coated with enteric polymer. Coating was performed in fluidized bed coater under conditions as listed in Table 2. Eudragit L 30 D is a 30% aqueous dispersion of anionic polymer Eudragit L dissolving at pH 5.5 or above due to carboxylic groups contained in the polymer. Coating was applied by fluidized bed coater under parameters listed in Table 2. Dissolution studies were carried out in USP apparatus I (basket) using 900 ml of 0.1 N HCl for 2 h and thereafter phosphate buffer pH 6.8. For coated pellets no release (or less than 10%) was observed at pH 1.2 (0.1 N HCl) for all weight gain samples and after that a rapid dissolution of pellets was observed. Since drug is highly water soluble and pellets contains 80% of drug. Therefore when coating solution was sprayed on to the pellets, it solubilized the drug and pulled it into the film as a result of which correct dissolution profile was not obtained. Hence it was decided to give a seal coat to protect the drug. After seal coat, Eudragit coat was applied up to 40%, 50% and 60% weight gain. Effect of different weight gain of acrylate polymer was evaluated by *in vitro* dissolution studies. The enteric properties as well as dissolution behavior of pellets could not altered by increasing the coat thickness to 60% (fig. 1). Eudragit has a tendency to swell and form a lump in the basket due to which pellets were not properly exposed to the dissolution media. Therefore, a 1% w/w film coat of glyceryl monostearate was applied over Eudragit coat, which prevented the aggregation of pellets in dissolution media. It was seen that the drug release from the coated pellets depended on the pH of the dissolution medium as well as weights applied. Pellet size and size distribution was determined for coated pellets with different weight gain and it was found to be narrow in distribution (fig. 2). Optimized pellets were evaluated for flow properties and friability as per the standard procedures and found to possess a good flow and friability (±0.5%) was within the permissible limit (±1%) (Table 5). Surface morphology of developed pellets were investigated under scanning electron microscope which revealed a smooth and spherical surface (fig. 3).

**TABLE 4:** EFFECT OF CERTAIN PARAMETERS ON PELLET SHAPE

Formulation	Parameters			Observation
		
	Spheronization speed (rpm)	Spheronization time (min)	Granulation liquid (ml)	
F1	1100	3	70	Dumbbell shaped
	1100	3	65	Rod shaped
	1100	3	75	Spherical in one lot and dumbbell in second lot.
	830	3	75	Dumbbell shaped
F2	1100	3	60	Dumbbell shaped
	1100	3	65	Dumbbell+Spherical
	1100	3	70	Dumbbell+Spherical
	830	3	70	Dumbbell shaped
F3	1100	3	60	Spherical shaped
	1100	1.5	65	Spherical shaped
	1100	2	65	Spherical shaped
	830	2	65	Dumbbell+spherical
F4	1100	3	60	Spherical shaped
	1100	1.5	65	Spherical shaped
	1100	2	65	Spherical shaped
	830	2	65	Spherical shaped

Effect of certain parameters such as speed and time of spheronization and granulation liquid used on the shape of pellets for formulations F1-F4

**TABLE 5:** EVALUATION CHARACTERISTICS OF UNCOATED PELLETS

Parameters	Mean Value (n=3, ±SD)
Pellets size (mm)	0.86 ± 0.13
Repose angle	12.58° ± 0.97
Aerated bulk density (g/ml)	0.767 ± 0.02
Packed bulk density (g/ml)	0.856 ± 0.04
Hausner’s ratio	1.11 ± 0.21
Carr’s index	10.39 ± 0.45
% friability	0.50% ± 0.07

The results indicated that it is possible to prevent the acidic degradation of sod PAS in upper GI tract which ultimately affect the bioavailability of drug and will help to reduce the dose, by development of multiparticulate system coated with pH dependent polymers using extrusion spheronization technique and fluidized bed processor.
